# Discovering pathway cross-talks based on functional relations between pathways

**DOI:** 10.1186/1471-2164-13-S7-S25

**Published:** 2012-12-07

**Authors:** Chia-Lang Hsu, Ueng-Cheng Yang

**Affiliations:** 1 Institute of Biomedical Informatics, National Yang-Ming University, Taipei, Taiwan; 2 Center for Systems and Synthetic Biology, National Yang-Ming University, Taipei, Taiwan; 3 Department of Life Science, National Taiwan University, Taipei, Taiwan

## Abstract

**Background:**

In biological systems, pathways coordinate or interact with one another to achieve a complex biological process. Studying how they influence each other is essential for understanding the intricacies of a biological system. However, current methods rely on statistical tests to determine pathway relations, and may lose numerous biologically significant relations.

**Results:**

This study proposes a method that identifies the pathway relations by measuring the functional relations between pathways based on the Gene Ontology (GO) annotations. This approach identified 4,661 pathway relations among 166 pathways from Pathway Interaction Database (PID). Using 143 pathway interactions from PID as testing data, the function-based approach (FBA) is able to identify 93% of pathway interactions, better than the existing methods based on the shared components and protein-protein interactions. Many well-known pathway cross-talks are only identified by FBA. In addition, the false positive rate of FBA is significantly lower than others via pathway co-expression analysis.

**Conclusions:**

This function-based approach appears to be more sensitive and able to infer more biologically significant and explainable pathway relations.

## Background

Pathway analysis is the currently best method for understanding the biological meanings of a set of genes derived from high-throughput experiments, such as gene expression microarray [1,2]. In biological systems, a pathway is a sequence of reactions or interactions among a subset of expressed genes related to a phenomenon or a biological process. Many methods, such as over-representation analysis [3] and gene set enrichment analysis (GSEA) [4], have been developed to identify the effective pathways for a given gene list. However, these methods may find numerous pathways that are independently represented. What is challenging is interpreting the significance of these pathways.

Pathways are not isolated entities in a cell, but may have cross-talks. In biology, the term "cross-talk" refers to the phenomenon that signal components in signal transduction can be shared between different signaling pathways [5]. Pathways coordinate or interact with one another in response to external stimuli, often having synergistic effects on certain biological processes. These interactions include sharing components, protein-protein interactions, and transcriptional regulations [5,6]. Perturbations on a pathway might affect the interacting pathways and comprehensively alter the phenotypes of a cell. Therefore, examining the interactions among pathways is essential for understanding the regulatory mechanisms of a given phenomenon.

Several computational approaches have been developed to identify pathway cross-talks. An intuitive method involves considering shared components between pathways [5,7,8]. Because pathway boundaries are arbitrary, related pathways may not share any components. Protein-protein interactions (PPIs) might mediate pathway interactions. Lu et al. [5] assessed pathway overlaps after extending a pathway with interacting proteins of pathway components. Li et al. [9] constructed a pathway cross-talk network (PCN) based on PPIs which connected between two pathways' components. The assumption is that if two pathways interact with each other, more PPIs are observed between these two pathways than expected. These methods, however, do not take into account that some genes in specific pathways may be not involved under particular condition. To address this, Huang and Li [10] and Liu et al. [11] incorporated gene expression profiles and PPIs to select active PPIs, and constructed phenotype-specific pathway cross-talk networks for angiogenesis and Alzheimer's disease, respectively.

Though these computational methods could determine the pathway cross-talks by p-values from different statistic methods, non-statistical significance may be biologically significant. In other words, cross-talking pathways may share only a few components or be connected by a few PPIs. For instance, the BMP and canonical WNT pathways in the Pathway Interaction Database (PID) [12] only share one component: GSK3B. In fact, these two pathways have been reported to possess biologically meaningful cross-talk [13]. Therefore, developing new approaches is required to detect cross-talk among pathways.

Two pathways interact with each other in order to participate or regulate a particular process for a specific condition. For instance, the cross-talk between the glucocorticoid receptor (GR) and T-cell antigen receptor (TCR) signaling pathways results in apoptosis during the development of thymocytes [14]. Additionally, activation of the GR triggers apoptosis in T cells, but activation of the TCR blocks GR-induced apoptosis [15]. It also implies that there is a functional cross-talk between these two distinct signaling systems. For another example, BMP and WNT signaling pathways are able to function independently from each other in numerous biological processes, such as stem cell differentiation, specification of cell fates, organogenesis, and carcinogenesis. However, in some conditions, they have to cross-talk to each other to cause effects, which cannot be achieved by either pathway individually [13]. Thus, if two pathways are implicated in many identical biological events, they may have high possibility to cross-talk in some conditions. In other words, we may be able to discover cross-talks in functionally related pathways.

In this study, we presented a function-based approach (FBA) to identify pathway cross-talks, measuring the functional similarity between pathways via the Gene Ontology (GO) annotations of pathway components. In our previous study this method has successfully been used to understand the functional relationship between RAS-regulated pathways [16]. Here, we extended this idea of pathway functional relations on discovering the pathway cross-talks.

## Results and discussion

### Analyzing pathways from single data source

There are many pathway databases available in public domains, such as KEGG [17], BioCarta, Reactome [18], GenMAPP [19], PID [12], and others. Because each pathway database has its own curation standard, a pathway event in different database may contain different components and interactions. For instance, mTOR signaling pathway is curated by many database, but a few components are common (Additional file 1). Therefore, to avoid redundancies, only the pathway data from a single database were used for analysis in this study.

Pathway data were downloaded from Pathway Interaction Database (PID) [12], which deposits curated and peer-reviewed human signaling and regulatory pathways. We chose PID for several reasons. Firstly, pathways in PID are curated by standardized criteria and reviewed by several curators, unlike BioCarta which pathways are contributed and uploaded by users. Secondly, PID separates a signaling event into two or more distinct pathways. For instance, WNT signals are transduced to different pathways for different situations. The canonical WNT pathway is through Frizzled (FZD) family receptors and LRP5/LRP6 co-receptors for cell fate determination, but the non-canonical WNT pathways through FZD family receptors and ROR2/RKY co-receptors for cell movement and tissue polarity [20]. Therefore, unlike KEGG which has only a WNT signaling pathway, PID deposits canonical and non-canonical WNT signaling pathways, respectively. Finally, PID also contains information of pathway cross-talks which can be used as positive data for evaluating the performance of different methods.

We collected 168 pathways from PID after removing redundant and less informative pathways (Additional file 2). Thus, there were 14,028 non-redundant pathway pairs. A function-based approach (FBA) was proposed to identify the pathway cross-talks from these pathway pairs.

### Using GO annotations to calculate the functional relations between pathways

Gene Ontology (GO) is widely used for functional annotations of genes. Most genes have been assigned with multiple GO terms, and the assignments of GO terms are generally based on pathways in which the genes participate. Therefore, the function annotations of a pathway could be inferred from the GO terms of the pathway components. The function-based approach (FBA) that we proposed applies this character to measure the functional similarities between pathways, comprising two steps: 1) inferring the representative GO terms for each pathway, and 2) calculating the similarity among pathways.

Though pathway components may find numerous relevant GO terms, these terms are not all suitable in describing the function of a given pathway. This problem is similar to the GO term enrichment analysis, which is conducted to investigate whether gene sets associated with particular GO terms. The Fisher exact test has been successfully used to identify "enriched" GO terms of a given gene set [3,21]. If all the genes in a gene set are from a single pathway, the enriched GO terms should sufficiently describe the function of this pathway. Thus, we employed the Fisher exact test to determine the representative GO terms of each pathway.

On average, 261 representative GO terms emerged per pathway (Additional file 3), covering 3,935 distinct GO terms. In these representative GO terms, many related descriptions and functions were correctly identified. Taking the WNT-mediated β-catenin pathway (PID pathway ID: wnt_beta_catenin_pathway) as an example is to examine if the related GO terms are correctly enriched. The WNT-mediated β-catenin pathway plays the role in regulation of cell proliferation and apoptosis and embryonic development [22,23]. In total 283 GO terms were selected as representative GO terms for the WNT-mediated β-catenin pathway. The relative terms, such as "regulation of Wnt receptor signaling pathway (GO: 0030111)", "regulation of cell proliferation (GO:0042127)", "regulation of apoptosis (GO:0042981)", and "mesoderm development (GO:0007498)", were correctly found. This example indicates the procedure of Fisher exact test infers the representative GO term of a pathway indeed.

In principle, functionally related pathways should share GO term annotations. Each pathway pair has on average 79 common GO terms (Additional file 3). Although the amount of overlap is tremendous, the information content of a GO term and the number of representative GO terms of a pathway should be taken into account to adjust and normalize the GO term overlap between pathways. We employed a Vector Space Model [24,25] to quantify the functional similarity between pathways. A weighting scheme associated with the importance of each GO term was determined by the frequency of this term occurring in annotations of the given pathway and whole human genes, and was incorporated into the vectors. Finally, a functional similarity score (*funSim*) between two pathways was computed via cosine measure. The higher the *funSim *score, the more functionally related this pathway pair.

### Identification of pathway cross-talk pairs

**Figure 1:**
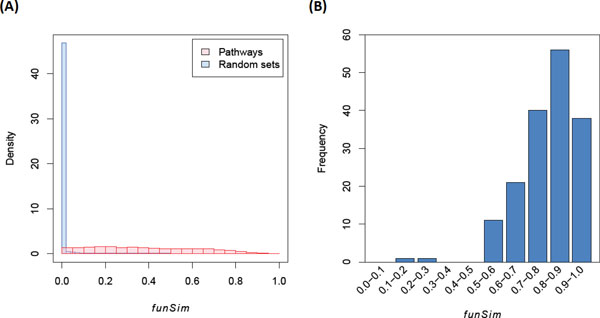
**Distributions of functional similarity (funSim) scores**. (A) The distribution of functional similarity scores of the pathway pairs and random sets, respectively. Each data point represents the fraction of the pathway pairs that have a *funSim *more than the value on the x-axis. (B) The distribution of the best *funSim *scores for all pathways.

The application of the aforementioned procedure calculated the functional similarity (*funSim*) score of the 14,028 combinations for 168 pathways. The distribution of these scores is shown in Figure 1A. Though different pathways may share numerous GO terms (Additional file 3), most of the pathway pairs have low scores.

In order to decide whether two pathways interact with each other, a cut-off value is required. The cut-off value was estimate by comparing the distribution of *funSim *of pathways with that of random gene sets (Figure 1A). To control the false positive rate as low as possible, the cut-off value of *funSim *was defined as 0.5, so that the false positive rate at this cutoff-value was less than 0.05%. Here, the pathway pairs which *funSim *are larger than the cut-off value denote as function-related pathway pairs (FRPs). However, the reliability of this cut-off value was further examined by two methods.

Firstly, the *funSim *were examined whether it can correctly reveal the functional relations. If two pathways are functionally related, they are more likely to fall into the same functional category. Therefore, the pathway pair from the same functional category should have higher *funSim *than those not in the same category. To test this hypothesis, pathways were manually classified into 8 categories based on the description of each pathway in PID. A pathway may be assigned into more than one category because a pathway may play multiple roles in a cell. Since 45 pathways lacked a clear description, only 123 pathways were classified (Additional file 4). For each functional category, the numbers of FRPs in inter- and intra-categories were counted, respectively, and the Fisher exact test was performed to access if these two values are occurred by chance (see Materials and Methods section for details). As shown in Table 1, in all categories, the proportion of FRPs in the same category (inter-category) is significantly higher than that not in the same category (intra-category). This result shows that *funSim *could distinguish whether pathways are functionally related.

**Table 1:** The results of functional category analysis

Category	# pathways	P-value*	R_e_^&^
Cell migration	35	3.32 × 10^-54^	3.672
Cell adhesion	31	1.19 × 10^-48^	4.038
Cytoskeleton organization	37	1.02 × 10^-34^	2.489
Immune response	27	6.96 × 10^-29^	3.211
Cell proliferation	34	6.34 × 10^-28^	2.444
Apoptosis	41	4.92 × 10^-15^	1.657
Development	43	4.51 × 10^-8^	1.394
Cell cycle	33	0.001	1.207

* The probability values are derived from Fisher exact test

^&^R_e _indicates the relative enrichment.

Secondly, since all pathways are part of regulator circuit in the cell, each pathway is expected to have at least one functionally related pathway. Thus, the largest *funSim *for each pathway should in principle larger than the cut-off value. As shown in Figure 1B, 166 out of 168 pathways support this argument. The only two pathways that failed to pass the cut-off value are the "effects of Botulinum toxin (botulinumtoxinpathway)" (max *funSim *= 0.15) and "circadian rhythm pathway (circadianpathway)" (max *funSim *= 0.29). The mechanism of both pathways may be different from other pathways. The "effect of Botulinum toxin", for example, is involved in the transportation of neuron transmitters and muscle contraction causes. Therefore, the mechanism and effects of this pathway are quite different from other signal transduction pathways.

On the whole, FBA can correctly identify the functional relations between pathways. Setting cut-off value of *funSim *as 0.5 is reliable to distinguish the functionally related pathway pairs. Based on this cut-off value, 4,661 out of 14,028 pathway pairs were functionally related pathway pairs (FRPs), which were among 166 pathways (Additional file 5). FRPs were considered as putative pathway cross-talk pairs.

### Comparison to other approaches

By re-implementing the analyses described by Li et al [9], the significantly overlapping pathway pairs (SOPs) and the significantly interacting pathway pairs (SIPs) were computed. For 14,028 pathway pairs, there were 2,412 SOPs, which were found by assessing the amount of shared components between pathways. The remaining 11,616 pathway pairs (*i.e*. non-SOPs) were further analyzed using protein-protein interactions (PPIs). Finally, 1,681 SIPs were identified. These two methods are complementary to each other, so they should be taken together to find all putative pathway cross-talk pairs, and we denote these two methods as the physical entity-based approach (PEBA). Therefore, PEBA identified 4,093 putative pathway cross-talk pairs among 167 pathways (Additional file 5).

**Figure 2:**
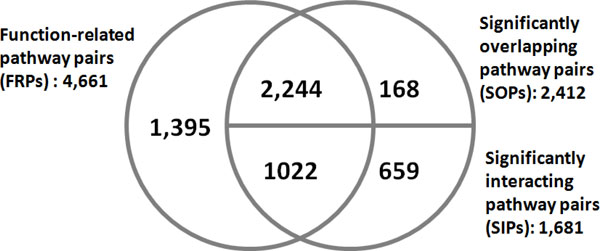
**Overlap of predicted pathway cross-talk pairs by different methods**. The different prediction methods were applied to all possible combinations of 168 pathways. FRPs are identified by the function-based approach (FBA), and SOPs and SIPs are determined by the physical entity-based approach (PEBA).

Figure 2 depicts the overlap between the predicted pathway cross-talks pairs of function-based approach (FBA) and physical entity-based approach (PEBA). There were 3,266 pathway pairs in common. Although FBA can identify 79.8% (3,266/4,093) of pathway pairs by PEBA, FBA and PEBA still have 1,395 and 827 unique pairs, respectively. This result shows that FBA and PEBA are substantially different on pathway cross-talk discovery.

To evaluate the performance of the different method, 143 pathway interacting or cross-talk pairs were extracted from PID as positive data sets. The performance of a method is evaluated in terms of precision and recall. Precision is the fraction of identified pathway pairs that are true pathway interactions. Recall is the fraction of true pathway interactions that are successfully identified. As shown in Table 2, although the precisions of both approaches are extremely low due to the limited size of positive data, FBA achieved recall of 0.93, higher than PEBA. The higher recall indicates that FBA is more sensitive than PEBA in discovering pathway cross-talks.

**Table 2:** Performance of different methods applied to 143 pathway interactions from PID

	FBA	PEBA
**Precision**	0.029 (134/4,661)	0.026 (106/4,093)
**Recall**	0.94(134/143)	0.74 (106/143)

Because PID does not comprehensively collect pathway interactions, many well-known pathway cross-talk pairs are not curated into PID. Table 3 presents additional 23 positive examples summarized from other methodology and review papers [26-29]. For instance, the pathway pairs of cxcr4_pathway to et_egfrpathway and vegfr1_pathway to et_egfrpathway are discussed in the SIP methodology paper [9]. FBA can identify 21 out of 24 well-known pathway cross-talks, but PEBA only identified 14 out of 24 pathway pairs. Although FBA is more sensitive to predict pathway interactions, a few well-studied pathway cross-talks are not detected by FBA, but by PEBA. For example, the cross-talk between glucocorticoid receptor (reg_gr_pathway) and FGF signaling pathway (fgf_pathway) was identified by PEBA as SOP, but the *funSim *of these two pathways be FAB is only 0.45 which is less than the cut-off of 0.5. Additionally, the pathway pairs of notch_pathway to tgfbrpathway and wnt_canonical_pathway failed to be identified by both FBA and PEBA (Table 3).

**Table 3:** List of well-documented pathway cross-talks

Pathway ID 1	Pathway ID 2	FBA	PEBA	PMID
erbb2erbb3pathway	vegfr1_2_pathway	0.86	SOP	19029832
fgf_pathway	erbb1_receptor_proximal_pathway	0.86	SOP	21045207
s1p_s1p1_pathway	vegfr1_2_pathway	0.78	SOP	20555359
s1p_s1p3_pathway	erbb1_receptor_proximal_pathway	0.78	SOP	20555359
fgf_pathway	pdgfrapathway	0.76	SOP	21045207
wnt_beta_catenin_pathway	vegfr1_2_pathway	0.73	NA	19806668
vegfr1_2_pathway	mapktrkpathway	0.71	SIP	18852899
fgf_pathway	mapktrkpathway	0.70	SIP	21045207
fgf_pathway	et_egfrpathway	0.68	SOP	21045207
vegfr1_pathway	et_egfrpathway	0.67	SIP	19357226
s1p_s1p1_pathway	pdgfrbpathway	0.67	SOP	20555359
fgf_pathway	pi3kciaktpathway	0.64	NA	21045207
erbb_network_pathway	et_egfrpathway	0.63	SOP	19223981
vegfr1_2_pathway	tgfbrpathway	0.60	SOP	19180561
hedgehog_2pathway	wnt_beta_catenin_pathway	0.60	NA	20085802
notch_pathway	hedgehog_2pathway	0.59	NA	17317139
cxcr4_pathway	et_egfrpathway	0.58	SIP	17601710
tcr_pathway	reg_gr_pathway	0.57	NA	8888490
hedgehog_2pathway	fgf_pathway	0.50	NA	20085802
hedgehog_2pathway	tgfbrpathway	0.50	NA	20085802
notch_pathway	vegfr1_2_pathway	0.50	NA	19273260
fgf_pathway	reg_gr_pathway	0.45*	SOP	21045207
notch_pathway	wnt_canonical_pathway	0.23*	NA	17317139
notch_pathway	tgfbrpathway	0.20*	NA	17317139

NA indicates this pathway pair is not identified by PEBA.

* indicates the value is less than cut-off value (*funSim *= 0.5).

To show that the abovementioned examples are not isolated cases, BMP signaling pathway (bmppathway) was chosen as a positive control to study its related pathways. This pathway is known to play diverse functions in vertebrates [30-32] and has cross-talk with numerous pathways to regulate a wide variety of biological process [33]. FBA identified 14 pathways that may have cross-talks with the BMP signaling pathway (Table 4). The pathways, including TGFβ, ALK1, ALK2, NOTCH, EGF, WNT, and SMAD2/3 signaling pathways, have been reported that they have cross-talks with the BMP pathway [13,33]. FBA also detected glypican signaling pathways (glypican_3pathway and glypican_1pathway) as related pathways. Current studies indicate that the main function of glypicans, heparan sulfate proteoglycans on the cell surface, consists in regulating several signaling pathways, including the BMP pathway [34]. However, PEBA identified total of 5 pathways related to cross-talk with BMP pathway, but only the NK-κB pathway (nfkappabcanonicalpathway), which might be activated by the BMP pathway for immune cytokine response [35], was not identified by FBA (Table 4).

**Table 4:** List of potential cross-talk pathways with BMP signaling pathway (bmppathway)

Pathway ID	FBA	PEBA
alk1pathway	0.75	SOP
tgfbrpathway	0.69	SOP
p38_mkk3_6pathway	0.59	NA
glypican_3pathway	0.55	NA
alk2pathway	0.58	SOP
ps1pathway	0.58	NA
wnt_noncanonical_pathway	0.57	NA
wnt_beta_catenin_pathway	0.53	NA
smad2_3nuclearpathway	0.53	NA
erbb1_receptor_proximal_pathway	0.53	NA
glypican_1pathway	0.51	NA
smad2_3pathway	0.51	SOP
erbb1_downstream_pathway	0.50	NA
wnt_canonical_pathway	0.50	NA
nfkappabcanonicalpathway	0.46*	SIP

* indicates the value is less than cut-off value (*funSim *= 0.5).

NA indicates this pathway pair is not identified by PEBA.

To summarize the results from these three different testing examples, it appears that FBA has the ability to discover pathway cross-talks. Moreover, FBA can identify the most documented pathway interactions. Because of the lack of negative data, the false positive rate of each approach cannot be evaluated. Here, an alternative evaluation, pathway co-expression analysis, was applied to assess the false positive rate.

### Assessment of false positive rate by pathway co-expression analysis

In a biological point of view, a pair of pathway can interact with each other if they are expressed at the same time and in the same place, *e.g*. tissue. Thus, pathway co-expression is a requisite for a real pathway cross-talk. This character can be used to evaluate the false positive rate of pathway cross-talk prediction methods: if the co-expression of a predicted pathway pair does not behave well, this pair may be a false positive.

Although the protein expression data are the best evidence to support co-expression, such data are difficult to obtain. This study used gene expression as an alternative, because a gene needs to be expressed before the protein is expressed. The expression of a pathway in a given tissue was assessed by the presence of expressed sequence tags (ESTs) for the components in this pathway. If two pathways are expressed in more common tissue, it implies these two pathways may have higher probability of being cross-talks. Here, the co-expression value of a pair of pathway was presented by the arithmetic average of Jaccard coefficient and overlap coefficient (see Methods section for details).

**Figure 3:**
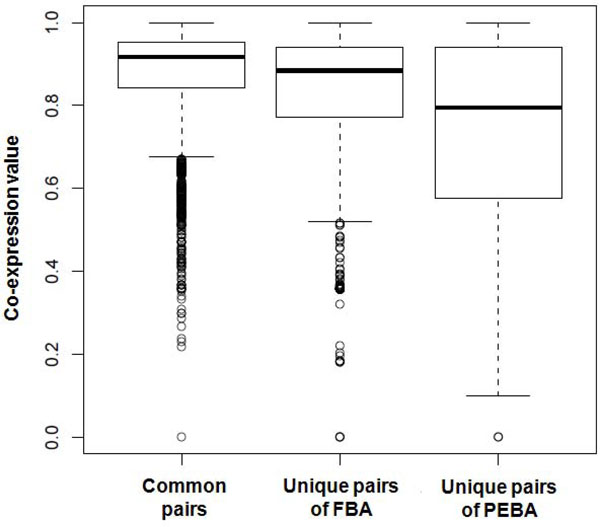
**Comparison of co-expression values among sets of pathway pairs inferred by different methods**. The distributions of coexpression values were compared among common pathway pairs, which are identified by FBA and PEBA, unique pairs by FBA, and unique pairs by PEBA, respectively.

The distributions of co-expression values of putative pathway cross-talk pairs are given in Figure 3. The common pairs identified by both of FBA and PEBA have the significantly higher co-expression values than unique pairs by FBA and PEBA, respectively (P-value < 2.2 × 10^-16^, Wilcoxon signed-rank test). This is not surprising, since the common pairs that pass the criteria of the two distinct approaches are the most reliable pathway cross-talk pairs. Interestingly, the average co-expression value of unique pairs of FBA is significantly higher than that of PEBA (P-value = 5.8 × 10^-12^, Wilcoxon signed-rank test). This result shows that the predicted pairs by FBA are more reliable than those by PEBA. In other words, the false positive rate of FBA might be less than that of PEBA.

### From pathway associations to cross-talk mechanisms

The previous results reveal that FBA is advantageous in discovering real pathway cross-talks. Furthermore, FBA can assist researchers to find some pathways associations which mechanisms are explainable. First, we focused on the pathway pairs which are predicted as FRPs and have only one common gene. Although these cases are unable to pass the criterion of SOPs, but this single shared component may be important for pathway interaction. Additionally, this shared component could assist us to understand the regulating relations between pathways without additional information.

**Figure 4:**
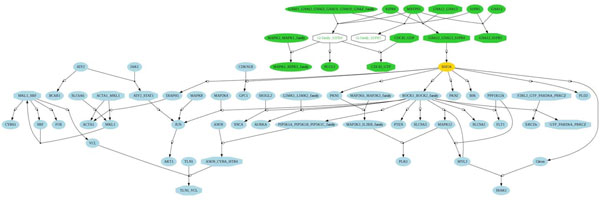
**Interaction between S1P4 and RHOA signaling pathways**. The pathways are integrated and visualized via the Pathway Integration Tool (PINT) [36]. The oval nodes denote the proteins, and the octagon nodes denote the protein complexes. The edges between nodes represent the interactions or reactions. The green and blue nodes correspond to the components in S1P4 and ROHA signaling pathways, respectively. The yellow node is the common component between pathways.

There were 2,301 pathway pairs with one common gene. In these pairs, 399 and 329 pairs are FRPs and SIPs, respectively, and 172 pairs are both FRPs and SIPs. We examined the 227 unique pathway pairs which are only identified by FBA and found several pathway interactions which are biologically significant. The first example is the pathway pair of S1P4 pathway (s1p_s1p4_pathway) and RHOA signaling pathway (rhoa_pathway). These two pathways share only one gene, *i.e*. RHOA, and are connected by 20 PPIs which is less than expected value. Hence, this pair is unable to pass the criteria of PEBA. However, FBA predicted this pair is highly function-related with *funSim *of 0.65. Merging these two pathways by the Pathway Integration Tool (PINT) [36] shows that the S1P4 pathway appears as the upstream of the RHOA signaling pathway (Figure 4). Sphingosine 1-phosphate (S1P) is a signaling lipid that plays a significant role in the regulation of cell growth, survival, and migration [27]. S1P might bind to S1P receptor 4 (S1P4) before regulating the activity of RHOA for particular biological processes. Numerous studies have reported that S1P stimulates cell motility through ROHA activation [27].

Another similar example is the interaction between syndecan-1-mediated signaling pathway (syndecan_1_pathway) and TGFβ receptor signaling pathway (tgfbrpathway). Though these two pathways share only one gene, *i.e*. TFGB1, and are connected by 41 PPIs, they do not satisfy the criteria of PEBA. However, they have a significantly high functional similarity score (*funSim *= 0.69), implying these two pathways should interact with each other. Indeed, this pathway association has been observed in different situations. In epithelial cells, syndecan-1 expression is regulated via TGFβ-mediated signaling [37]. In cardiac fibrosis, syndican-1 may promote TGFβ activation or activate the downstream TGFβ signaling pathways [38]. These evidences strongly suggest that these two pathways may interact with each other for different biological processes.

**Figure 5:**
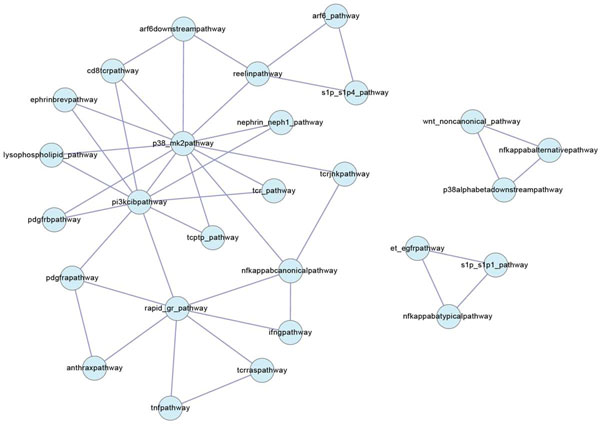
**Pathway cross-talk cliques**. The nodes indicate the pathway, and the edges are the pathway pairs which have no common gene and are not SIPs, but are identified as highly functional relations by FBA.

If a pathway pair does not share any component (*i.e*. non-SOP), and the number of PPIs between this pair fails to pass the statistical test (*i.e*. non-SIP), this pathway interactions is easy to ignore by PEBA. Therefore, FBA is able to re-connect the relations of these pathway pairs. Total of 200 FRPs among 104 pathways are unable to be detected by PEBA and without common genes. Among these pathway interactions, several pathways formed cliques, which is a set of pathways fully connected to each other. We used CFinder [39] to identify pathway crosstalk cliques. 17 size-3 cliques were present among 32 pathways identified (Figure 5). Interestingly, 7 out of 17 cliques contains the interaction between p38 mediated by MAPK (p38_mk2pathway) and class IB PI3K (pi3kcibpathway) signaling pathways. Indeed, the cross-talk between these two pathways involves in varied biological process [40,41]. Additionally, some cliques have been demonstrated the biological significance. For example, cross-talks among class IB PI3K (pi3kcibpathway), p38 mediated by MAPK (p38_mk2pathway), and PDGFRβ (pdgfrbpathway) pathways are associated with the differentiation of neural stem cells [42] and proliferation of vascular smooth muscle cells [43]. For another example, the TCR mediated by JNK (tcrjnkpathway), p38 mediated by MAPK (p38_mk2pathway), and canonical NF-κB (nfkappabcanonicalpathway) pathways are influenced each other in T cell activation [44].

Such "pathway cross-talk cliques" also imply the complexity of regulations. For example, the pathway cross-talk clique of S1P1 (s1p_s1p1_pathway), atypical NF-κB (nfkappabatypicalpathway), and EGFR (et_egfrpathway) pathways (Figure 5) reveals the two ways to regulate one pathway. The association of S1P1 and EGFR has been well documented that S1P1 pathway activates the EGFR signaling in cancer cell [45]. However, S1P1 might also activate EGFR pathway through the NF-κB pathway. This phenomenon has been observed in rat vascular smooth muscle cells in which S1P1 induce EGFR expression via NF-κB pathway [46]. This case also demonstrates the pathway interaction information is useful for understanding the regulatory mechanisms.

### Assessing reproducibility of cross-talks pairs by FBA

Because there are many pathway databases available, we examined if FBA can produce the same results by using different pathway data. We manually collected corresponding signaling events from PID and Reactome, respectively. The ten signaling events which are all curated by PID and Reactome, respectively, were analyzed and listed in Table 5. There were 45 possible combinations for 10 pathways. We found no signaling event curated by two databases is completely identical, and the average size of pathway components in PID is larger than that of Reactome. Nonetheless, we expected that the FBA can produce the consistent results from different data sets.

**Table 5:** Pathways used to assess the reproducibility

Pathway	# components in PID	# components in Reactome	# common components
BMP signaling pathway	42	23	17
EGFR signaling pathway	35	52	16
TGFβ signaling pathway	55	15	10
NOTCH signaling pathway	37	16	15
FGFR signaling pathway	55	45	26
PDGFR signaling pathway	56	65	20
VEGFR signaling pathway	63	11	8
p38 MAPK signaling pathway	27	13	2
mTOR signaling pathway	70	27	11
Insulin pathway	45	108	17

**Figure 6:**
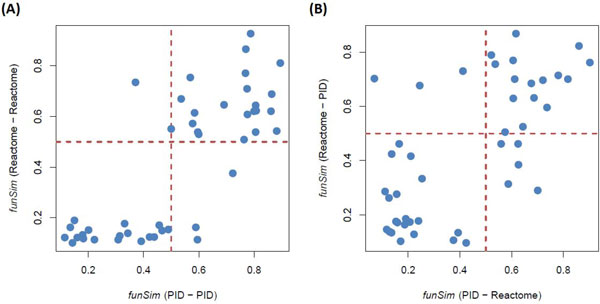
**Reproducibility analysis of function-based approach**. The left figure (A) is the results of using pathway pairs from the same database, and the right figure (B) is the results of using pathway pairs cross databases. Each data point represents *funSim *score of a pathway pair. The dash lines represent the cut-off value (*funSim *= 0.5). If the points are located in the left-bottom and right-top corners, the prediction results of these pathway pairs are consistent. In contrast, the points located in the left-top and right-bottom corners indicate the inconsistent results.

Firstly, the *funSim *scores of pathway pairs from the same database were performed. As shown in Figure 6A, the *funSim *score between using pathways from PID and Reactome is highly correlated (Pearson correlation coefficient = 0.78). When cut-off value of 0.5 was used to distinguish if the two pathways interact with each other, 41 out of 45 pathway pairs are consistent between two pathway databases. The four inconsistent cases are the pathway pairs PDGFR-mTOR, mTOR-TGFβ, VEGFR-TGFβ, and PDGFR-p38 MAPK. The first three cases are the *funSim *> 0.5 by using PID data and *funSim *< 0.5 by using Reactome, but the last one is opposite.

Secondly, the *funSim *scores of pathway pairs from different databases, e.g. for each pathway pairs, one pathway is from PID, and the other is from Reactome, were calculated. The *funSim *scores for PID-Reactome (*i.e*. the first pathway from PID and the second one from Reactome) and Reactome-PID (*i.e*. the first pathway from Reactome and the second one from PID) reveal still high correlation (Pearson correlation coefficient = 0.67) (Figure 6B). Only 8 out of 45 pathway pairs resulted in different conclusions. The five cases which *funSim *of PID-Reactome is larger than 0.5, but *funSim *of Reactome-PID is less than 0.5 are pathway pair FGFR-p38 MAPK, insulin-p38 MAPK, BMP-TGFβ, PDGFR-p38 MAPK, and TGFβ-p38 MAPK. Additionally, the three cases, which funSim of PID-Reactome are less than 0.5, but funSim of Reactome-PID are larger than 0.5, are pathway pairs FGFR-VEGFR, PDGFR-mTOR, and VEGFR-p38 MAPK.

The results show that prediction of pathway cross-talks by FBA is robust and reproducible even though the pathways curated by different databases are diverse. Additionally, the *funSim *of 0.5 can be a general threshold to determine the pathway cross-talks.

### Limitation of FBA for prediction of pathway cross-talks

Despite the fact that FBA has the ability to predict the pathway cross-talks, it does have limitations. Because the relations are inferred from the GO annotations of pathway, the performance of FBA may be dependent on the quality of GO annotations. In this study, Fisher exact test with a multiple testing correction was applied to selection of the representative GO terms of a pathway. However, this procedure might still infer the false pathway-GO term associations due to the quality of GO annotations of genes [47] and the intrinsic limitations of statistical analysis [3]. The former one might be improved by considering the evidence codes of GO annotations which has been used in this study. The latter one might be overcome by the advanced statistical models [48,49]. Moreover, in the portion of similarity measurement, we did not consider the relations between GO terms and simply compute the proportion of common terms by cosine measure. Therefore, FBA might be further improved by the advanced GO semantic similarity measurement techniques [50,51].

Some important pathway interactions still fail to be identified by these computational approaches. The notable example is NOTCH signaling pathway (notch_pathway). This pathway is important to developmental process and able to integrate with several major pathways [29]. However, FAB and PEBA only identified 3 (syndecan_3_pathway, vegfr1_2_pathway, and hedgehog_2pathway) and 4 (syndecan_3_pathway, p75ntrpathway, ps1pathway, and il1pathway) pathways cross-talking with NOTCH pathway, respectively. Actually, some pathways, such as WNT and TGFβ signaling pathways, interacting with NOTCH pathway are essential for development [29], but these interactions are not detected by all approaches (Table 3). In addition, as shown in Figure 1B, "effect of Botulinum toxin" (botulinumtoxinpathway) and "circadian rhythm pathway" (circadianpathway) fail to be identified interacting pathways by FBA. These two pathways also fail to be detected interacting pathways by PEBA, except E2F transcription factor network (e2f_pathway) which was defined as significantly interacting with circadian rhythm pathway. All in all, computational approaches have still many limitations for predicting pathway cross-talks.

## Conclusions

We present a novel approach to identifying pathway cross-talks by measuring the functional relations between pathways. The functional relations rely on GO annotations of pathway components through the vector space model. The concept of this function-based approach (FBA) quite differs from the physical entity-based approach (PEBA) which identifies pathway cross-talks based on shared components and protein-protein interaction. The comparison with PEBA corroborates the contribution of FBA. Many well-studied pathway cross-talks can only be successfully predicted by FBA. Though FBA identified approximately 4,600 putative pathway interactions among 166 pathways, the false positive rate of FBA might be significantly less than that of PEBA, assessed by pathway co-expression analysis. Therefore, FBA not only is more sensitive to detect cross-talks but also infers more biologically significantly and explainable pathway relations. The most important character of FBA is that the analysis results are reproducible even though different pathway data were used. With the development of pathway analysis and visualization tools, this approach can assist biological researchers to propose a potential mechanism and to prioritize the pathways for further experimental design.

Undoubtedly, discovery of pathway cross-talks is indeed important for understanding biological regulations, but we may face several challenges. The first problem is the lack of gold standard data sets. Therefore, we are unable to evaluate the prediction power of a new method. In this study, we collected real pathway interactions from PID and literature. Although the collection is not comprehensive, it may be sufficient for evaluation of different methods. Additionally, we used an alternative way, considering the pathway co-expression, to assess the false positive rate. The second problem is that pathways are dynamic, not static. Pathway may interact with the other pathways in a specific condition, but dot not influence each other in another condition. Several studies address this problem by incorporating gene expression information to filter the active interactions. Finally, because each method has its own advantage on predicting certain pathway cross-talks, how to integrate different characters to improve the precision of prediction is an important task in the future.

## Materials and methods

### Pathway data

Pathway data containing 184 pathways with 2,346 genes were downloaded from the Pathway Interaction Database (PID) [12] in Aug. 2010. Because pathways with too few genes may not possess sufficient biological content for analysis, 13 pathways with less than five genes were removed [9]. Additionally, three pathways that entirely overlapped with other pathways were also ignored, because they may be redundant. Finally, 168 pathways with 2,285 genes were collected.

PID also records some pathway interaction information. These pathway interactions were extracted from XML format file of each pathway. Finally, 143 pathway interactions among 118 pathways were obtained from PID and regarded as positive data.

### Determining GO terms to represent a pathway

The proposed function-based approach (FBA) utilizes GO annotations to measure the functional similarity between pathways. The first step required determining the GO terms that could represent the biological function of a pathway. The GO terms were obtained from the GO website (http://www.geneontology.org/), and the GO annotations for all human genes were downloaded from NCBI Entrez Gene (ftp://ftp.ncbi.nlm.nih.gov/gene/DATA/gene2go.gz). Only GO terms from the biological process ontology were analyzed. To avoid an overestimation of the performance of our pathway cross-talk predicting method, only annotations based on directly experimental evidence were considered, including EXP (inferred from experiment), IDA (inferred from direct assay), IPI (inferred from physical interaction), IMP (inferred from mutant phenotype), IGI (inferred from genetic interaction), IEP (inferred from expression pattern), and IC (inferred by curator). In other words, these gene-GO term associations are supported by relevant literature.

The over-representation analysis method, a common method to assess the enrichment of specific biological themes in a gene list [3], was used to determine the representative GO terms of a pathway. A one-sided Fisher exact test was performed to evaluate whether GO terms are enriched in a pathway, and then the *P*-values were adjusted via the Benjamini-Hochberg (BH) method [52]. GO terms with adjusted *P*-values < 0.05 were considered representative GO terms for a pathway.

### Measurement of functional similarity between pathways

A vector space model was used to compute the functional similarity between pathways. A pathway is represented by a specific vector *p_i_*, as follows:

(1) $$p_{i}=\left(w_{i,1},w_{i,2},...,w_{i,n}\right)$$

where *w_i, j _*is the weight of the representative GO term *j *for pathway *i*, and *n *is the number of representative GO terms associated with all pathways. If the pathway does not have a given GO term, the weight of the GO term for this pathway is 0. Since not all GO terms are equally informative, the TF-IDF (term frequency-inverse document frequency measure, which is common used for information retrieval [53], was applied to determine the weight of each term. The concept is that the importance of a term increases proportionally to frequency of this term appearing in the pathway but is offset by the frequency of this term appearing in the whole genes in a given organism. Therefore, the weight of term *j *in pathway *i *is calculated as:

(2) $$w_{i,j}=tf_{i,j}*\text{log}\frac{N_{G}}{tf_{G,j}}$$

where *N_G _*is the total number of genes in a given organism *G*, and *tf_i, j _*and *tf_G, j _*are the frequency of genes annotated by term *j *in pathway *i *and a given organism *G*, respectively. Because GO is presented as a directed acyclic graph (DAG), a GO term's semantics inherits the biological meanings of all its parent terms. In other words, when a term is used to describe a gene, all its parent terms also apply to this gene. Therefore, the frequency of GO term *j *appearing in pathway *i, tf_i, j_*, is given by:

(3) $$tf_{i,j}=a_{i,j}+\sum_{t\in children(j)}a_{i,t}$$

where *a_i, j _*is the number of genes in pathway *i *annotated by the term *j*, and *children(j) *is the set of child terms of term *j*.

The functional similarity between pathways can be qualified by comparing the vectors. The functional similarity score of a pathway pair, *funSim(p_1_, p_2_)*, was calculated by using the cosine measure [54], defined as:

(4) $$funSim\left(p_{1},p_{2}\right)=\frac{{\overset{⇀}{p}}_{1}\cdot {\overset{⇀}{p}}_{2}}{\left|p_{1}\right|\left|p_{2}\right|}$$

The functional similarity was performed pairwisely for all pathways of the collections.

### Background distribution of *funSim*

The background distribution of *funSim *was estimated by a randomization procedure. 9,011 human genes were collected from NCBI Entrez after removing genes without annotations of GO terms from the biological process ontology. For each pathway, the number of annotated GO terms of each gene in a given pathway was counted and replaced by other gene which was randomly drawn from the set of 9,011 genes and has the same number of annotated GO terms. The random gene set list was generated, and the *funSim *of each pair of random gene sets was calculated. The background distribution of *fusSim *was established from the merged results of the 100 random gene set lists.

### Functional category analysis

The pathways were manually categorized into different functional groups based on the "biological process" descriptions which were extracted from XML format file of each pathway in PID. 8 functional categories were collected. Because a pathway may play multiple roles in a cell, a pathway may be assigned to multiple functional categories.

For each functional category, the proportions of function-related pathway pairs (FRPs) in intra- and inter-categories were compared. If a given functional category has *k *pathways, *N_intra _*and *N_inter _*pathway pairs are present in the intra- and inter-category, respectively, where *N_intra _*is the pairwise combination of *k *pathways (i.e., $\left(\begin{array}{c} k\\ 2 \end{array}\right)$), and *N_inter _*is the combination between *k *pathways and all pathways excluding pathways in this category. The number of FRPs, *n_intra _*and *n_inter_*, were then counted in the intra- and inter-category, respectively. The null hypothesis is that the ratio of *n_intra_/N_intra _*is the same as the ratio of *n_inter_/N_inter_*. Fisher exact test was used to assess the proportional differences of FPRs between the intra- and inter-category. In addition, relative enrichment, *R_e_*, was calculated and defined as follows:

(5) $$R_{e}=\frac{n_{intra}/N_{intra}}{n_{inter}/N_{inter}}$$

These two factors were used to evaluate the effect of the function-based method on measurement of functional similarity between pathways.

### Identifying pathway cross-talks by shared components

An intuitive manner of studying pathway cross-talks is by measuring the number of shared components between pathways. Li et al [9] utilized the Fisher exact test to assess whether the components of two pathways were significantly overlapping. The *P*-values were adjusted via BH method. The pathway pairs with adjusted *P*-value < 0.05 were regarded as the significantly overlapping pathway pairs (SOPs).

### Identifying pathway cross-talks via protein-protein interactions

The remaining pathway pairs that were not significantly overlapping (non-SOPs) were studied via protein-protein interactions (PPIs). PPIs were downloaded from QuasiPro (http://csb2.ym.edu.tw/quasipro), which integrated PPI data from nine sources, including DIP [55], BIND [56], IntAct [57], MIPS [58], MINT [59], HPRD [60], BioGRID [61], Reactome [18], and Pathway Commons [62]. This dataset contains 140,382 interactions and 12,164 human genes.

PPIs were used to assess the pathway cross-talks according to the procedure proposed by Li et al. [9]. The number of PPIs among components of each pathway pair was counted. An estimation of the background distribution of the protein-interaction count of each pathway pair was generated from 1,000 rounds of the randomization procedure, as described in Li's study [9]. A one-sided Fisher exact test was performed to assess whether two pathways are significantly interacting, and then *P*-values were adjusted via the BH method. Pathway pairs with adjusted *P*-value < 0.05 were considered significantly interacting pathway pairs (SIPs).

### Pathway co-expression analysis

The gene expression information was based on the ESTs (Expression Sequence Tags) from UniGene (Build 222). The standardized tissue names for libraries were obtained from the Cancer Genome Anatomy Project (CGAP, http://cgap.nci.nih.gov/Tissues/). 46 tissues were present in the EST dataset, excluding uncharacterized and pooled tissues. ESTs from the same tissues were pooled together. A gene is considered to be expressed in a specific tissue if at least one of its EST sequences is found in the dataset.

For each pathway and tissue, an assessment was conducted on whether the pathway was expressed in a given tissue. A p-value was calculated by the hypergeometric test:

(6) $$p_{}=\overset{n}{\underset{i=k}{\sum }}\frac{\left(\begin{array}{c} m\\ i \end{array}\right)\left(\begin{array}{c} N-m\\ n-i \end{array}\right)}{\left(\begin{array}{c} N\\ n \end{array}\right)}$$

where *N *is the number of total genes in the human genome, *m *is the number of expressed genes in a given tissue, *n *is the number of components in a given pathway, and *k *is the number of pathway components expressed in this tissue. If a pathway with *P*-value < 0.05 in a given tissue, this pathway is regarded as expressed pathway in this tissue.

The co-expression value between two pathways was measured by combining Jaccard coefficient (*JC*) and overlap coefficient (*OC*) defined as:

(7) $$JC=\frac{E_{12}}{E_{1}+E_{2}-E_{12}}\;\text{and}\;OC=\frac{E_{12}}{\text{min}\left(E_{1},E_{2}\right)}$$

Where *E_1 _*and *E_2 _*denote the number of tissues in which pathway 1 and 2 are expressed, respectively, and *E_12 _*denotes the number of common tissues where both pathways are expressed. Because both coefficients have weakness when *E_1 _*and *E_2 _*are imbalance, the arithmetic average of *JC *and *OC *was used to refer to as the co-expression value between two pathways here.

## Competing interests

The authors declare that they have no competing interests.

## Authors' contributions

CLH conceived, designed, and performed the experiments. UCY analyzed and interpreted data. All the authors wrote and revised the manuscript.

## Supplementary Material

Additional File 1 **Curation of mTOR signaling pathway in different databases**. mTOR signaling pathway in BioCarta (A), KEGG (B), and PID (C), respectively, are curated with different components (D). Click here for file

Additional File 2 **List of PID pathways used in this study**. Click here for file

Additional File 3 **Enriched GO terms for each pathway**. (A) Distribution of the number of enriched GO terms per pathway. (B) Distribution of the number of common GO terms per pathway pair. Click here for file

Additional File 4 **Manually curated functional categories**. Click here for file

Additional File 5 **List of putative pathway cross-talks predicted by FBA and PEBA**. Click here for file
