# Supplementary figures and images for: Discovering pathway cross-talks based on functional relations between pathways
Source: BMC Genomics. 2012 Dec 7;13(Suppl 7):S25. doi: 10.1186/1471-2164-13-S7-S25 (PMC3521217; doi:10.1186/1471-2164-13-S7-S25)

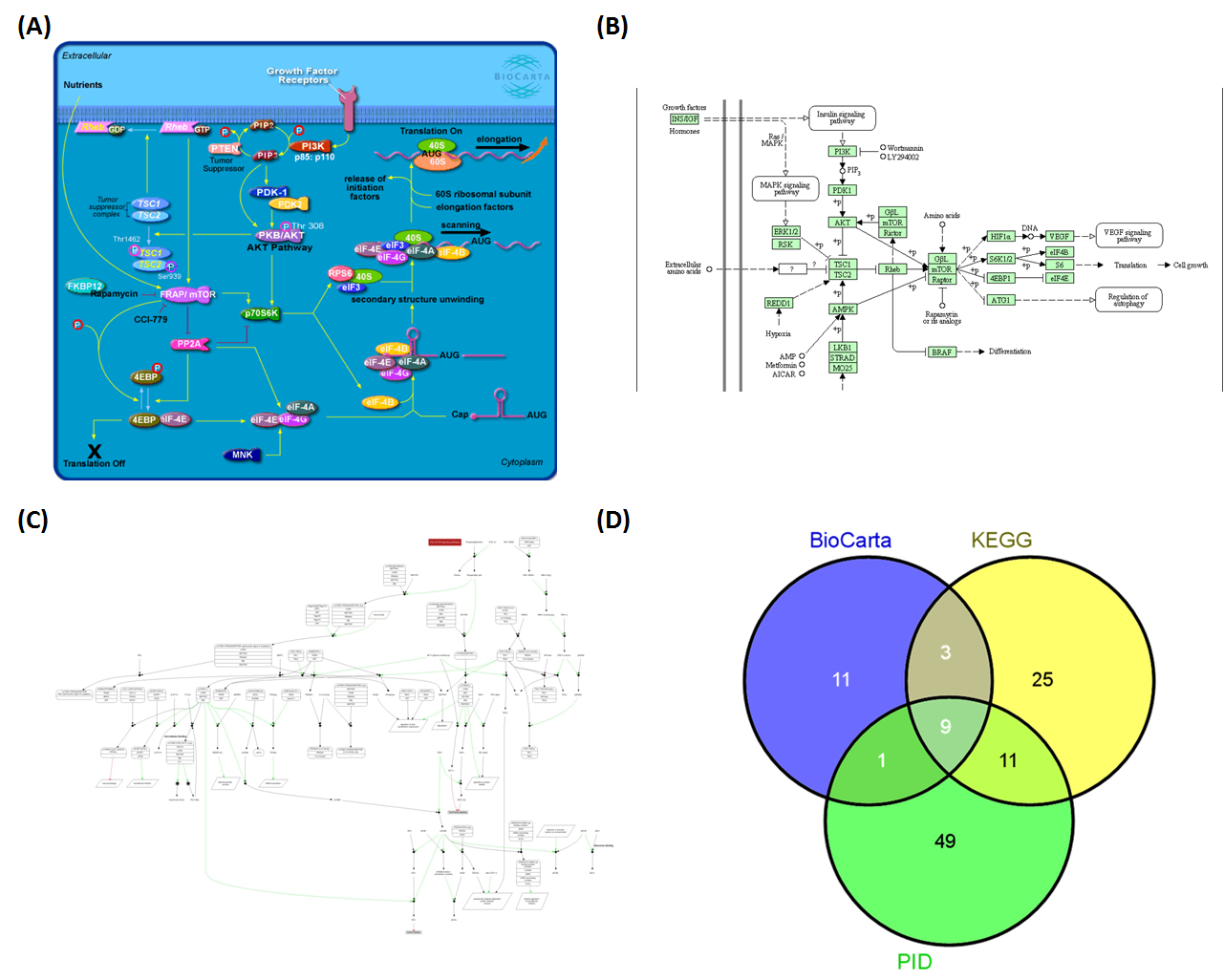

Supplement: Additional File 1 — Curation of mTOR signaling pathway in different databases. mTOR signaling pathway in BioCarta (A), KEGG (B), and PID (C), respectively, are curated with different components (D). [file 1471-2164-13-S7-S25-S1.png]

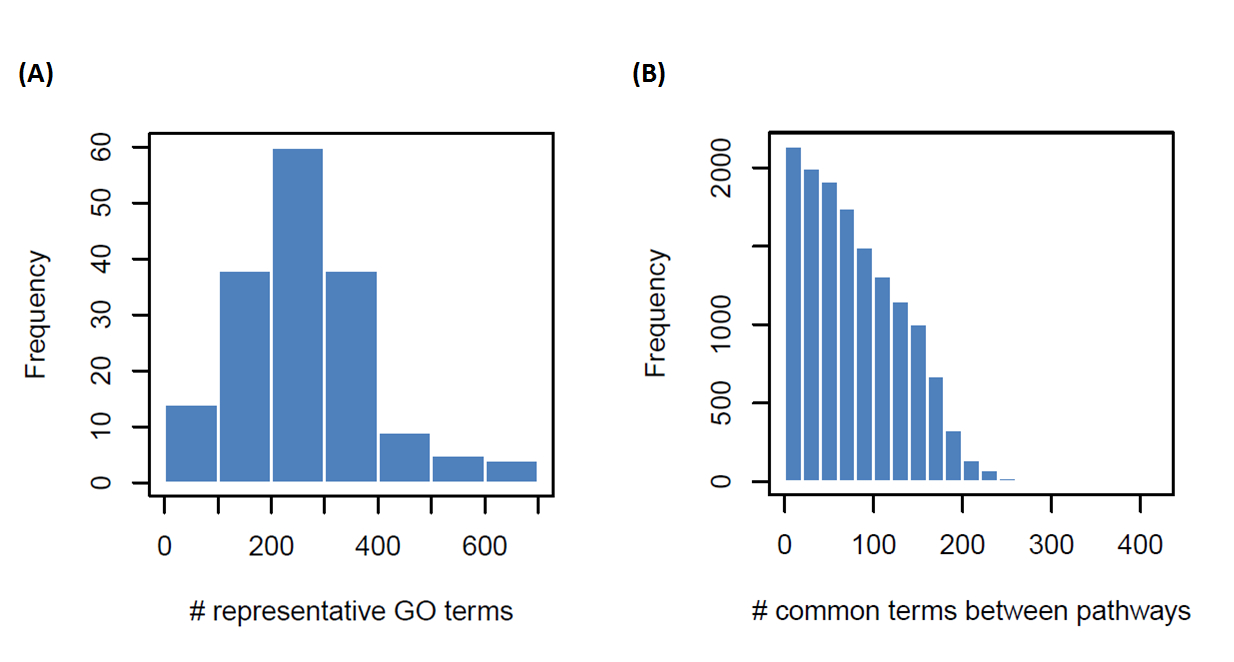

Supplement: Additional File 3 — Enriched GO terms for each pathway. (A) Distribution of the number of enriched GO terms per pathway. (B) Distribution of the number of common GO terms per pathway pair. [file 1471-2164-13-S7-S25-S3.png]
