# Supplementary material for: Discovering pathway cross-talks based on functional relations between pathways
Source: BMC Genomics. 2012 Dec 7;13(Suppl 7):S25. doi: 10.1186/1471-2164-13-S7-S25 (PMC3521217; doi:10.1186/1471-2164-13-S7-S25)
Supplement: Additional File 2 — List of PID pathways used in this study. [file 1471-2164-13-S7-S25-S2.pdf]

Additional file 2 – List of pathway from PID

Pathway ID	Pathway name	# components	# enriched GO terms
a4b1_paxdep_pathway	Paxillin-dependent events mediated by a4b1	20	179
a4b1_paxindep_pathway	Paxillin-independent events mediated by a4b1 and a4b7	24	213
a6b1_a6b4_integrin_pathway	a6b1 and a6b4 Integrin signaling	46	243
alk1pathway	ALK1 signaling events	26	544
alk2pathway	ALK2 signaling events	11	105
alphasynuclein_pathway	Alpha-synuclein signaling	33	225
amb2_neutrophils_pathway	amb2 Integrin signaling	61	233
angiopoietinreceptor_pathway	Angiopoietin receptor Tie2-mediated signaling	50	502
anthraxpathway	Cellular roles of Anthrax toxin	19	331
ar_nongenomic_pathway	Nongenotropic Androgen signaling	31	225
ar_pathway	Coregulation of Androgen receptor activity	61	128
ar_tf_pathway	Regulation of Androgen receptor activity	53	164
arf_3pathway	Arf1 pathway	18	99
arf6_pathway	Arf6 signaling events	35	205
arf6_traffickingpathway	Arf6 trafficking events	50	58
arf6downstreampathway	Arf6 downstream pathway	16	181
atf2_pathway	ATF-2 transcription factor network	50	491
aurora_a_pathway	Aurora A signaling	31	161
aurora_b_pathway	Aurora B signaling	40	65
avb3_integrin_pathway	Integrins in angiogenesis	49	414
avb3_opn_pathway	Osteopontin-mediated events	32	341
bard1pathway	BARD1 signaling events	29	265
bcr_5pathway	BCR signaling pathway	66	327
bmppathway	BMP receptor signaling	42	509
botulinumtoxinpathway	Effects of Botulinum toxin	9	74
caspase_pathway	Caspase cascade in apoptosis	52	115

cd40_pathway	CD40/CD40L signaling	31	300
cd8tcrdownstreampathway	Downstream signaling in naive CD8+ T cells	66	269
cd8tcrpathway	TCR signaling in naive CD8+ T cells	53	336
cdc42_pathway	CDC42 signaling events	70	237
cdc42_reg_pathway	Regulation of CDC42 activity	30	66
ceramide_pathway	Ceramide signaling pathway	48	491
circadianpathway	Circadian rhythm pathway	14	17
cmyb_pathway	C-MYB transcription factor network	84	244
cone_pathway	Visual signal transduction: Cones	23	22
cxcr3pathway	CXCR3-mediated signaling events	43	239
cxcr4_pathway	CXCR4-mediated signaling events	100	244
e2f_pathway	E2F transcription factor network	74	220
ecadherin_keratinocyte_pathway	E-cadherin signaling in keratinocytes	22	332
ecadherin_nascentaj_pathway	E-cadherin signaling in the nascent adherens junction	40	260
ecadherin_stabilization_pathway	Stabilization and expansion of the E-cadherin adherens junction	42	201
endothelinpathway	Endothelins	64	369
epha_fwdpathway	EPHA forward signaling	34	102
epha2_fwdpathway	EPHA2 forward signaling	19	184
ephbfwdpathway	EPHB forward signaling	40	200
ephrinbrevpathway	Ephrin B reverse signaling	28	192
epopathway	EPO signaling pathway	34	222
er_nongenomic_pathway	Plasma membrane estrogen receptor signaling	41	231
erbb_network_pathway	ErbB receptor signaling network	15	152
erbb1_downstream_pathway	ErbB1 downstream signaling	108	520
erbb1_internalization_pathway	Internalization of ErbB1	41	200
erbb1_receptor_proximal_pathway	EGF receptor (ErbB1) signaling pathway	35	225
erbb2erbb3pathway	ErbB2/ErbB3 signaling events	44	269
erbb4_pathway	ErbB4 signaling events	38	265

et_egfrpathway	EGFR-dependent Endothelin signaling events	9	339
fak_pathway	Signaling events mediated by focal adhesion kinase	59	233
faspathway	FAS (CD95) signaling pathway	38	223
fcer1pathway	Fc-epsilon receptor I signaling in mast cells	62	234
fgf_pathway	FGF signaling pathway	55	232
foxm1pathway	FOXM1 transcription factor network	41	271
foxopathway	FoxO family signaling	49	253
glypican_1pathway	Glypican 1 network	27	654
glypican_3pathway	Glypican 3 network	8	366
hdac_classi_pathway	Signaling events mediated by HDAC Class I	66	321
hdac_classii_pathway	Signaling events mediated by HDAC Class II	34	273
hdac_classiii_pathway	Signaling events mediated by HDAC Class III	26	268
hedgehog_2pathway	Signaling events mediated by the Hedgehog family	22	121
hedgehog_glipathway	Hedgehog signaling events mediated by Gli proteins	48	131
hif1_tfpathway	HIF-1-alpha transcription factor network	66	376
hif1apathway	Hypoxic and oxygen homeostasis regulation of HIF-1-alpha	19	162
hif2pathway	HIF-2-alpha transcription factor network	34	196
hivnefpathway	HIV-1 Nef: Negative effector of Fas and TNF-alpha	35	271
hnf3apathway	FOXA1 transcription factor network	43	123
hnf3bpathway	FOXA2 and FOXA3 transcription factor networks	45	270
ifngpathway	IFN-gamma pathway	41	263
igf1_pathway	IGF1 pathway	30	294
il12_2pathway	IL12-mediated signaling events	63	375
il12_stat4pathway	IL12 signaling mediated by STAT4	33	304
il1pathway	IL1-mediated signaling events	34	266
il2_1pathway	IL2-mediated signaling events	55	296
il2_pi3kpathway	IL2 signaling events mediated by PI3K	36	318
il2_stat5pathway	IL2 signaling events mediated by STAT5	30	251

il23pathway	IL23-mediated signaling events	37	500
il27pathway	IL27-mediated signaling events	26	614
il4_2pathway	IL4-mediated signaling events	65	243
il6_7pathway	IL6-mediated signaling events	48	411
insulin_glucose_pathway	Insulin-mediated glucose transport	27	257
insulin_pathway	Insulin Pathway	45	350
kitpathway	Signaling events mediated by Stem cell factor receptor (c-Kit)	52	254
lis1pathway	Lissencephaly gene (LIS1) in neuronal migration and development	29	63
lkb1_pathway	LKB1 signaling events	47	177
lpa4_pathway	LPA4-mediated signaling events	15	132
lymphangiogenesis_pathway	VEGFR3 signaling in lymphatic endothelium	24	265
lysophospholipid_pathway	LPA receptor mediated events	67	377
mapktrkpathway	Trk receptor signaling mediated by the MAPK pathway	34	200
met_pathway	Signaling events mediated by Hepatocyte Growth Factor Receptor (c-Met)	80	379
mtor_4pathway	mTOR signaling pathway	70	343
myc_activpathway	Validated targets of C-MYC transcriptional activation	79	229
myc_pathway	C-MYC pathway	25	181
myc_represspathway	Validated targets of C-MYC transcriptional repression	63	360
ncadherinpathway	N-cadherin signaling events	34	203
nectin_pathway	Nectin adhesion pathway	30	317
nephrin_neph1_pathway	Nephrin/Neph1 signaling in the kidney podocyte	32	282
nfat_3pathway	Role of Calcineurin-dependent NFAT signaling in lymphocytes	55	233
nfat_tfpathway	Calcineurin-regulated NFAT-dependent transcription in lymphocytes	48	327
nfkappabalternativepathway	Alternative NF-kappaB pathway	6	86
nfkappabatypicalpathway	Atypical NF-kappaB pathway	17	329
nfkappabcanonicalpathway	Canonical NF-kappaB pathway	23	454
notch_pathway	Regulation of Notch signaling	37	98
p38_mk2pathway	p38 signaling mediated by MAPKAP kinases	21	194

p38_mkk3_6pathway	p38 MAPK signaling pathway	27	207
p38alphabetadownstreampathway	Signaling mediated by p38-alpha and p38-beta	38	207
p38alphabetapathway	Regulation of p38-alpha and p38-beta	31	248
p38gammadeltapathway	Signaling mediated by p38-gamma and p38-delta	11	111
p53downstreampathway	Direct p53 effectors	138	422
p53regulationpathway	p53 pathway	59	317
p75ntrpathway	p75(NTR)-mediated signaling	69	246
pdgfrapathway	PDGFR-alpha signaling pathway	25	318
pdgfrbpathway	PDGFR-beta signaling pathway	56	272
pi3kciaktpathway	Class I PI3K signaling events mediated by Akt	35	274
pi3kcibpathway	Class IB PI3K non-lipid kinase events	6	110
pi3kcipathway	Class I PI3K signaling events	56	210
pi3kplctrkpathway	Trk receptor signaling mediated by PI3K and PLC-gamma	36	225
plk1_pathway	PLK1 signaling events	46	210
prlsignalingeventspathway	Signaling events mediated by PRL	23	171
ps1pathway	Presenilin action in Notch and Wnt signaling	46	317
ptp1bpathway	Signaling events mediated by PTP1B	51	369
rac1_pathway	RAC1 signaling pathway	54	145
rac1_reg_pathway	Regulation of RAC1 activity	40	89
ranbp2pathway	Sumoylation by RanBP2 regulates transcriptional repression	11	188
rapid_gr_pathway	Rapid glucocorticoid signaling	8	151
rb_1pathway	Regulation of retinoblastoma protein	65	390
reelinpathway	Reelin signaling pathway	29	218
reg_gr_pathway	Glucocorticoid receptor regulatory network	82	408
ret_pathway	Signaling events regulated by Ret tyrosine kinase	40	293
retinoic_acid_pathway	Retinoic acid receptors-mediated signaling	30	385
rhoa_pathway	RhoA signaling pathway	42	145
rhoa_reg_pathway	Regulation of RhoA activity	46	107

rhodopsin_pathway	Visual signal transduction: Rods	24	46
rxr_vdr_pathway	RXR and RAR heterodimerization with other nuclear receptor	26	393
s1p_meta_pathway	Sphingosine 1-phosphate (S1P) pathway	21	65
s1p_s1p1_pathway	S1P1 pathway	21	314
s1p_s1p2_pathway	S1P2 pathway	24	167
s1p_s1p3_pathway	S1P3 pathway	29	279
s1p_s1p4_pathway	S1P4 pathway	14	112
smad2_3nuclearpathway	Regulation of nuclear SMAD2/3 signaling	82	304
smad2_3pathway	Regulation of cytoplasmic and nuclear SMAD2/3 signaling	18	167
syndecan_1_pathway	Syndecan-1-mediated signaling events	17	168
syndecan_2_pathway	Syndecan-2-mediated signaling events	33	326
syndecan_3_pathway	Syndecan-3-mediated signaling events	17	94
syndecan_4_pathway	Syndecan-4-mediated signaling events	33	373
tcptp_pathway	Signaling events mediated by TCPTP	42	370
tcr_pathway	TCR signaling in naive CD4+ T cells	66	350
tcrcalciumpathway	Calcium signaling in the CD4+ TCR pathway	30	134
tcrjnkpathway	JNK signaling in the CD4+ TCR pathway	14	152
tcrraspathway	Ras signaling in the CD4+ TCR pathway	14	181
telomerasepathway	Regulation of Telomerase	68	617
tgfbrpathway	TGF-beta receptor signaling	55	681
tnfpathway	TNF receptor signaling pathway	46	275
trail_pathway	TRAIL signaling pathway	28	210
trkrpathway	Neurotrophic factor-mediated Trk receptor signaling	63	229
txa2pathway	Thromboxane A2 receptor signaling	58	260
vegfr1_2_pathway	Signaling events mediated by VEGFR1 and VEGFR2	63	448
vegfr1_pathway	VEGFR1 specific signals	28	339
wnt_beta_catenin_pathway	Regulation of Wnt-mediated beta catenin signaling and target gene transcription	80	283
wnt_canonical_pathway	Canonical Wnt signaling pathway	31	353

wnt_noncanonical_pathway	Noncanonical Wnt signaling pathway	33	398
wnt_signaling_pathway	Wnt signaling network	28	336
