# Supplementary material for: Discovering pathway cross-talks based on functional relations between pathways
Source: BMC Genomics. 2012 Dec 7;13(Suppl 7):S25. doi: 10.1186/1471-2164-13-S7-S25 (PMC3521217; doi:10.1186/1471-2164-13-S7-S25)
Supplement: Additional File 4 — Manually curated functional categories. [file 1471-2164-13-S7-S25-S4.pdf]

Additional file 4 – Manually curated function categories

Category	Pathways	"Biological process" descriptions in PID
**Cell migration**	avb3_integrin_pathway	cell migration
	amb2_neutrophils_pathway	cell migration
	fcer1pathway	positive regulation of cell migration
	smad2_3nuclearpathway	endothelial cell migration
	erbb1_downstream_pathway	cell migration
	syndecan_4_pathway	cell migration
	erbb4_pathway	neural crest cell migration
	lysophospholipid_pathway	cell migration
		endothelial cell migration
	erbb2erbb3pathway	cell migration
	angiopoietinreceptor_pathway	cell migration
	wnt_noncanonical_pathway	cell migration
	epha_fwdpathway	cell migration
	faspathway	cell migration
	kitpathway	cell migration
	s1p_s1p1_pathway	endothelial cell migration
	vegfr1_2_pathway	cell migration
	a6b1_a6b4_integrin_pathway	cell migration
	alk1pathway	endothelial cell migration
	syndecan_3_pathway	positive regulation of leukocyte migration
	pdgfrbpathway	cell migration
	arf6downstreampathway	regulation of epithelial cell migration
	syndecan_1_pathway	keratinocyte migration
		positive regulation of cell migration
	ephrinbrevpathway	endothelial cell migration
	ptp1bpathway	cell migration
	erbb1_receptor_proximal_pathway	cell migration
	s1p_s1p3_pathway	endothelial cell migration
	avb3_opn_pathway	endothelial cell migration
	ephbfwdpathway	endothelial cell migration
		cell migration
	epha2_fwdpathway	endothelial cell migration
	s1p_s1p4_pathway	cell migration
	lymphangiogenesis_pathway	cell migration
	reelinpathway	neuron migration
	fgf_pathway	cell migration
	fak_pathway	cell migration
	cdc42_pathway	cell migration
**Cell adhesion**	avb3_integrin_pathway	cell adhesion
		regulation of cell-matrix adhesion
	amb2_neutrophils_pathway	leukocyte adhesion
	nephrin_neph1_pathway	heterophilic cell-cell adhesion
		homophilic cell adhesion
	syndecan_1_pathway	regulation of cell adhesion

		positive regulation of cell-cell adhesion
		positive regulation of cell migration
	arf6downstreampathway	regulation of calcium-dependent cell-cell adhesion
	ephrinbrevpathway	cell adhesion
	syndecan_4_pathway	cell adhesion
		positive regulation of cell adhesion
	a4b1_paxdep_pathway	cell adhesion
	cxcr4_pathway	regulation of heterotypic cell-cell adhesion
		cell adhesion
	il2_1pathway	positive regulation of cell-cell adhesion
	rhoa_pathway	focal adhesion formation
		cell adhesion mediated by integrin
	cxcr3pathway	cell adhesion
	angiopoietinreceptor_pathway	focal adhesion formation
	ncadherinpathway	regulation of cell-cell adhesion
	a4b1_paxindep_pathway	positive regulation of cell-cell adhesion
		cell adhesion
	epha_fwdpathway	beta-integrin mediated adhesion
		cell-cell adhesion
	ecadherin_stabilization_pathway	regulation of cell-cell adhesion
		regulation of calcium-dependent cell-cell adhesion
	epopathway	beta-integrin mediated adhesion
	avb3_opn_pathway	cell adhesion
	ephbfwdpathway	cell-cell adhesion
	epha2_fwdpathway	cell-cell adhesion
		cell adhesion mediated by integrin
	arf6_traffickingpathway	cell adhesion mediated by integrin
		regulation of calcium-dependent cell-cell adhesion
	ecadherin_keratinocyte_pathway	homophilic cell adhesion
	reelinpathway	neuron adhesion
	er_nongenomic_pathway	cell adhesion
	a6b1_a6b4_integrin_pathway	cell adhesion
	nectin_pathway	cell adhesion
	fak_pathway	positive regulation of cell adhesion
	syndecan_2_pathway	positive regulation of cell-cell adhesion
	ecadherin_nascentaj_pathway	regulation of calcium-dependent cell-cell adhesion
		regulation of cell-cell adhesion
		regulation of heterotypic cell-cell adhesion
	syndecan_3_pathway	positive regulation of cell-cell adhesion
**Cytoskeleton organization**	amb2_neutrophils_pathway	actin filament polymerization
	ar_nongenomic_pathway	actin cytoskeleton reorganization
	caspase_pathway	actin filament polymerization
	bcr_5pathway	actin cytoskeleton organization
	fcer1pathway	actin cytoskeleton reorganization

	lis1pathway	negative regulation of microtubule depolymerization
		actin polymerization or depolymerization
	erbb1_downstream_pathway	cytoskeleton organization
		tight junction assembly
		lamellipodium assembly
	syndecan_4_pathway	cytoskeleton organization
	rhoa_pathway	actin filament depolymerization
	cxcr3pathway	actin polymerization or depolymerization
	epha_fwdpathway	actin filament depolymerization
	txa2pathway	actin cytoskeleton reorganization
	kitpathway	actin filament polymerization
	ecadherin_stabilization_pathway	adherens junction organization
		cortical actin cytoskeleton stabilization
		actin cytoskeleton organization
		cortical microtubule organization
	tcr_pathway	actin polymerization or depolymerization
	ecadherin_keratinocyte_pathway	adherens junction organization
		actin cable formation
	vegfr1_2_pathway	actin cytoskeleton reorganization
	syndecan_2_pathway	actin cytoskeleton reorganization
	arf_3pathway	actin filament polymerization
	pdgfrbpathway	actin cytoskeleton reorganization
	nephrin_neph1_pathway	cytoskeleton organization
	arf6downstreampathway	cortical actin cytoskeleton organization
		actin filament bundle formation
	p38_mk2pathway	actin cytoskeleton reorganization
	mtor_4pathway	regulation of actin cytoskeleton organization
	cxcr4_pathway	actin filament depolymerization
	erbb1_receptor_proximal_pathway	cortical actin cytoskeleton organization
	plk1_pathway	microtubule cytoskeleton organization
		positive regulation of microtubule depolymerization
	ncadherinpathway	actin filament polymerization
		adherens junction organizatio
	pdgfrapathway	actin cytoskeleton reorganization
	pi3kcipathway	actin cytoskeleton reorganization
	ephbfwdpathway	actin cytoskeleton reorganization
	il2_pi3kpathway	actin cytoskeleton organization
	fgf_pathway	cytoskeleton organization
	rac1_pathway	actin filament depolymerization
		actin filament polymerization
	ecadherin_nascentaj_pathway	actin cytoskeleton organization
	cdc42_pathway	actin filament depolymerization
		actin cytoskeleton organization
	arf6_traffickingpathway	clathrin coat assembly

**Cell proliferation**	ar_nongenomic_pathway	cell proliferation
	avb3_integrin_pathway	cell proliferation
	erbb1_downstream_pathway	cell proliferation
	erbb4_pathway	cell proliferation
	cxcr3pathway	cell proliferation
	vegfr1_pathway	endothelial cell proliferation
	erbb2erbb3pathway	cell proliferation
	il4_2pathway	T-helper 2 cell differentiation
		T cell proliferation
	angiopoietinreceptor_pathway	cell proliferation
	txa2pathway	cell proliferation
	kitpathway	cell proliferation
		positive regulation of cell proliferation
	epopathway	cell proliferation
	il2_stat5pathway	cell proliferation
	il27pathway	T cell proliferation
	s1p_s1p1_pathway	negative regulation of T cell proliferation
	vegfr1_2_pathway	endothelial cell proliferation
	alk1pathway	endothelial cell proliferation
	cd40_pathway	B cell proliferation
	pdgfrbpathway	cell proliferation
	il23pathway	T cell proliferation
		keratinocyte proliferation
	ephrinbrevpathway	endothelial cell proliferation
	il2_1pathway	cell proliferation
		T cell proliferation
	cxcr4_pathway	cell proliferation
	il12_2pathway	T cell proliferation
	ar_pathway	cell proliferation
	anthraxpathway	regulation of endothelial cell proliferation
	ncadherinpathway	endothelial cell proliferation
	pdgfrapathway	cell proliferation
	cd8tcrdownstreampathway	alpha-beta T cell proliferation
	ceramide_pathway	cell proliferation
	il2_pi3kpathway	cell proliferation
		T cell proliferation
	lymphangiogenesis_pathway	cell proliferation
	ar_tf_pathway	cell proliferation
	cdc42_pathway	cell proliferation
**Immune response**	amb2_neutrophils_pathway	leukocyte activation during inflammatory
		immune response cell surface activating receptor
	tcrjnkpathway	immune response
	il23pathway	positive regulation of chronic inflammatory response
		positive regulation of humoral immune response
		positive regulation of T cell mediated cytotoxicity

	ifngpathway	Antibacterial Response
		Antiviral Response
	fcer1pathway	regulation of mast cell degranulation
		negative regulation of mast cell degranulation
		cytokine secretion
	bcr_5pathway	cytokine secretion
	cd8tcrpathway	cytotoxic T cell degranulation
	il12_2pathway	T cell proliferation
		natural killer cell mediated cytotoxicity
	tcrraspathway	immune response
	nfkappabalternativepathway	regulation of B cell activation
	anthraxpathway	inflammatory response
		negative regulation of macrophage activation
	kitpathway	immune response
	nfat_tfpathway	T cell activation
		T cell anergy
		CD4-positive CD25-positive alpha-beta regulatory T cell lineage commitment
	il2_stat5pathway	activation-induced cell death of T cells
		CD4-positive CD25-positive alpha-beta regulatory T cell lineage commitment
	il27pathway	T cell proliferation during immune response
		cytokine production during immune response
		mast cell activation
	cd8tcrdownstreampathway	cytotoxic T cell degranulation
		alpha-beta T cell proliferation
	tcr_pathway	immune response
	tcrcalciumpathway	immune response
	telomerasepathway	immune response
	nfat_3pathway	activation-induced cell death of T cells
	p38alphabetapathway	positive regulation of innate immune response
	il12_stat4pathway	T-helper 1 cell differentiation
		T-helper 2 cell differentiation
		natural killer cell mediated cytotoxicity
	angiopoietinreceptor_pathway	immune response
	cd40_pathway	B cell proliferation
		B cell affinity maturation
	il2_1pathway	T cell proliferation
		natural killer cell mediated cytotoxicity
	il2_pi3kpathway	T cell proliferation
	il4_2pathway	T-helper 2 cell differentiation
		T cell proliferation
		T-helper 1 cell differentiation
**Apoptosis**	ar_nongenomic_pathway	apoptosis
	avb3_integrin_pathway	apoptosis
	caspase_pathway	DNA fragmentation during apoptosis
		apoptotic nuclear changes

		apoptosis
		nuclear fragmentation during apoptosis
		neuron apoptosis
	ifngpathway	apoptosis
	erbb1_downstream_pathway	apoptosis
	erbb4_pathway	apoptosis
	erbb2erbb3pathway	apoptosis
	nfkappabatypicalpathway	positive regulation of anti-apoptosis
		cell death
	faspathway	apoptosis
	hdac_classiii_pathway	apoptosis
	glypican_1pathway	cell death
	il2_stat5pathway	activation-induced cell death of T cells
	telomerasepathway	DNA damage response signal transduction by p53 class mediator resulting in induction of apoptosis
	ecadherin_keratinocyte_pathway	apoptosis
	il1pathway	apoptosis
	er_nongenomic_pathway	apoptosis
	a6b1_a6b4_integrin_pathway	apoptosis
	nfat_3pathway	apoptosis
	hif2pathway	neuron apoptosis
	reg_gr_pathway	apoptosis
	trkrpathway	neuron apoptosis
	nephrin_neph1_pathway	apoptosis
	met_pathway	apoptosis
	igf1_pathway	apoptosis
	tnfpathway	apoptosis
	p53regulationpathway	apoptosis
	anthraxpathway	apoptosis
	ncadherinpathway	apoptosis
	mapktrkpathway	neuron apoptosis
	nfat_tfpathway	apoptosis
	pi3kplctrkpathway	neuron apoptosis
	ceramide_pathway	apoptosis
		cell survival
	alphasynuclein_pathway	cell death
		neuron apoptosis
	ps1pathway	apoptosis
	fgf_pathway	negative regulation of apoptosis
	p75ntrpathway	neuron apoptosis
	fak_pathway	apoptosis
	p38alphabetapathway	apoptosis
	glypican_3pathway	apoptosis
	trail_pathway	cell death
	il12_stat4pathway	apoptosis

**Development**	pdgfrapathway	oligodendrocyte development
	trkrpathway	neuron projection morphogenesis
		Schwann cell development
	hedgehog_glipathway	embryonic digit morphogenesis
		embryonic limb morphogenesis
		forebrain development
	arf6downstreampathway	tube morphogenesis
		liver development
	erbb4_pathway	dendrite morphogenesis
		neuron projection morphogenesis
		epithelial cell differentiation
		heart development
		glial cell differentiation
	rac1_pathway	neuron projection development
	notch_pathway	Bergmann glial cell differentiation
		skeletal muscle tissue development
		oligodendrocyte development
	glypican_1pathway	neuron differentiation
	glypican_3pathway	skeletal system development
		embryonic digit morphogenesis
		kidney development
	hedgehog_2pathway	heart development
		mesenchymal cell differentiation
		pancreas development
		dorsoventral neural tube patterning
		skeletal system development
	hif2pathway	germ cell development
	angiopoietinreceptor_pathway	vasculogenesis
		tube development
	erbb2erbb3pathway	nervous system development
		heart morphogenesis
		mammary gland morphogenesis
	faspathway	neuron projection development
	cdc42_pathway	neuron projection development
		endothelial cell morphogenesis
	ret_pathway	tube development
		neurite development
	pi3kplctrkpathway	axonogenesis
	smad2_3nuclearpathway	mesenchymal cell differentiation
		muscle cell differentiation
	tcptp_pathway	macrophage differentiation
	il4_2pathway	T-helper 2 cell differentiation
	ncadherinpathway	osteoblast differentiation
		axonogenesis
		myoblast differentiation
	hdac_classiii_pathway	muscle cell differentiation

	kitpathway	megakaryocyte differentiation
	ecadherin_stabilization_pathway	epithelial cell differentiation
	nfat_tfpathway	T-helper 1 cell differentiation
		T-helper 2 cell differentiation
	il27pathway	T-helper 2 cell differentiation
	il2_1pathway	T cell differentiation
	il6_7pathway	macrophage differentiation
	ecadherin_keratinocyte_pathway	keratinocyte differentiation
	reelinpathway	neuron differentiation
	rb_1pathway	fat cell differentiation
		erythrocyte differentiation
	cd40_pathway	plasma cell differentiation
	il12_stat4pathway	T-helper 1 cell differentiation
		T-helper 2 cell differentiation
	lymphangiogenesis_pathway	lymphangiogenesis
		vasculogenesis
	ephrinbrevpathway	neuron projection morphogenesis
	epha_fwdpathway	neuron projection morphogenesis
	txa2pathway	cell morphogenesis
	mapktrkpathway	neuron projection morphogenesis
	ephbfwdpathway	neuron projection morphogenesis
	epha2_fwdpathway	neuron projection morphogenesis
		angiogenesis
	p75ntrpathway	neuron projection morphogenesis
	syndecan_2_pathway	dendrite morphogenesis
		angiogenesis
	syndecan_3_pathway	neuron projection morphogenesis
**Cell cycle**	myc_represspathway	cell cycle arrest
	ar_nongenomic_pathway	regulation of S phase of mitotic cell cycle
	bcr_5pathway	re-entry into mitotic cell cycle
	prlsignalingeventspathway	mitosis
		G1/S transition of mitotic cell cycle
	bard1pathway	G1/S transition of mitotic cell cycle
	aurora_b_pathway	spindle checkpoint
	il2_1pathway	G1/S transition of mitotic cell cycle
	plk1_pathway	G2/M transition of mitotic cell cycle
	tcptp_pathway	G1/S transition of mitotic cell cycle
		positive regulation of S phase of mitotic cell cycle
	angiopoietinreceptor_pathway	negative regulation of cell cycle
	faspathway	cell cycle
	circadianpathway	S phase of mitotic cell cycle
	hdac_classiii_pathway	regulation of S phase of mitotic cell cycle
	il2_stat5pathway	G1/S transition of mitotic cell cycle
	telomerasepathway	cell cycle
	il6_7pathway	cell cycle arrest

	ceramide_pathway	negative regulation of cell cycle
	e2f_pathway	cell cycle
	rac1_pathway	regulation of cell cycle
	rb_1pathway	cell cycle
	p75ntrpathway	cell cycle arrest
	fak_pathway	regulation of cell cycle
	ecadherin_nascentaj_pathway	positive regulation of S phase of mitotic cell cycle
	aurora_a_pathway	G2/M transition checkpoint
		G2/M transition of mitotic cell cycle
	pi3kciaktpathway	negative regulation of cell cycle
		G1/S transition of mitotic cell cycle
	cdc42_pathway	regulation of cell cycle
	pdgfrbpathway	cell cycle arrest
	lis1pathway	cell cycle
	lymphangiogenesis_pathway	cell cycle
	p38_mk2pathway	G2/M transition checkpoint
	p38gammadeltapathway	G2/M transition checkpoint
	p53downstreampathway	G2/M transition DNA damage checkpoint
	ecadherin_keratinocyte_pathway	cell cycle
